# Income-Related Inequalities in Access to Dental Care Services in Japan

**DOI:** 10.3390/ijerph14050524

**Published:** 2017-05-12

**Authors:** Akemi Nishide, Misuzu Fujita, Yasunori Sato, Kengo Nagashima, Sho Takahashi, Akira Hata

**Affiliations:** 1 Department of Public Health, Chiba University Graduate School of Medicine, Chiba 260-8670, Japan; akeminishide@chiba-u.jp (A.N.); ahata@faculty.chiba-u.jp (A.H.); 2 Department of Global Clinical Research, Chiba University Graduate School of Medicine, Chiba 260-0870, Japan; yasu@faculty.chiba-u.jp (Y.S.); nshi@chiba-u.jp (K.N.); 3 Clinical Research Center, Chiba University Hospital, Chiba 260-0870, Japan; sho@chiba-u.jp

**Keywords:** socioeconomic status, access to dental care services, inequality

## Abstract

*Background*: This study aimed to evaluate whether income-related inequalities in access to dental care services exist in Japan. *Methods*: The subjects included beneficiaries of the National Health Insurance (NHI) in Chiba City, Japan, who had been enrolled from 1 April 2014 to 31 March 2015. The presence or absence of dental visits and number of days spent on dental care services during the year were calculated using insurance claims submitted. Equivalent household income was calculated using individual income data from 1 January to 31 December 2013, declared for taxation. *Results*: Of the 216,211 enrolled subjects, 50.3% had dental care during the year. Among those with dental visits, the average number of days (standard deviation) spent on dental care services per year was 7.7 (7.1). Low income was associated with a decreased rate of dental care utilization regardless of age and sex. However, there was a significant inverse linear association between the number of days spent on dental care services and income levels for both sexes. *Conclusions*: There were income-related inequalities in access to dental care services, regardless of the age group or sex, within the Japanese universal health insurance system.

## 1. Introduction

Socioeconomic inequalities in oral health have been reported in a number of countries [1,2,3,4]; a decreased proportion of individuals with 20 or more teeth was observed in a low-income group of Norwegians aged 25–79 years [1] and Japanese aged 65 years or older [2]. One meta-analysis showed that low socioeconomic status (SES) is associated with a higher prevalence of dental caries [3]. Although the mechanism behind the link between poor oral health and low SES has not been fully elucidated, lack of access to dental care services in low SES groups is considered one of the causes of oral health inequality [5]. In fact, associations between lower SES and decreased access to dental services have been found in several countries [6,7,8,9,10,11]. However, a comparative study investigating access to dental services among European countries detected a different impact of SES, probably due to the impact of country-specific health insurance systems [8]. To date, in Japan, only one study has reported on the association between SES and access to dental services [7]; this study showed a significant income-related inequality in access to preventive dental care services, but not curative care services, among 3083 subjects aged 25–50 years. The small sample size and selection bias due to the low response rate of 31.3% were considered limitations in this study [7]. Thus, knowing whether income-related inequality exists in Japan where universal insurance systems have been established would be interesting.

All Japanese people, except for those on public assistance for whom medical services are provided free of charge, have to obtain public health insurance. There are two major public health insurances for individuals under 75 years of age: the Employees’ Health Insurance for employees of the government and companies, and the National Health Insurance (NHI) for self-employed workers including farmers and fishers, retirees, or the unemployed [12]. For all elderly individuals aged 75 years or older, the Long Life Health Insurance has been established. The health insurance system in Japan runs on an age-dependent benefit system. At the time of this survey, the insured age groups of 0–6 years, 7–69 years, and 70 years or older were billed 20%, 30%, and 10–30% of their total medical fees, respectively. Moreover, the insurance provides an income- and age-dependent capped maximum copayment system for high-cost medical care [12]. Chiba City, a satellite urban city of Tokyo and the target area of this study, also implements a medical fee reduction policy for children and adolescents under the age of 15. Under this policy, any outpatient medical bill, which includes dental, is limited to 300 yen per visit for the residents aged 0–8 years, and 500 yen for those aged 9–15 years [13]. Under the universal health care insurance system, patients are able to choose any medical facility they want to visit [12].

Our objective was to evaluate whether the association between income level and access to dental care services existed among individuals aged from 0 to 74 years, enrolled on NHI in Chiba City, Japan.

## 2. Materials and Methods

### 2.1. Subjects

This study was a retrospective cohort study using individual income data obtained from tax records and the insurance claims for dental care administrated by the Chiba City. The subjects were beneficiaries of NHI (National Health Insurance) in Chiba City, Japan, who had been enrolled on the insurance from 1 April 2014 to 31 March 2015.

### 2.2. Ethics Statement

As this was a retrospective, an observational cohort study using existing data collated by the Chiba City Hall, consent was not obtained from each subject enrolled in this study. Since investigators had a contract with Chiba City Hall prohibiting the disclosure of any data provided without written consent, the dataset used for this study was not made openly available. The study protocol was approved by the Research Ethics Committee of the Graduate School of Medicine, Chiba University (Approval number 1724). The study was carried out in accordance with the principals of the Declaration of Helsinki and Ethical Guidelines for Medical and Health Research Involving Human Subjects.

### 2.3. Access to Dental Care Services

Access to dental care services was assessed by using insurance claim data from the NHI in Chiba City submitted from April 2014 to March 2015. Claims were excluded if they lacked information on individual identification. Subjects were defined as users (≥1 claim for dental care services) and non-users (no claims for dental care services) for dental care services. Furthermore, we determined the number of days spent for the services by totaling the number of days recorded in the claims for dental treatments for each individual.

### 2.4. Equivalent Household Income

Individual income data from 1 January to 31 December 2013 were obtained from tax records at the Chiba City Hall. Simply stated, equivalent household income was calculated by dividing the household income to the square root of the total number of household members [14]. Details of the method to determine household income and number of household members have been described elsewhere [13]. Equivalent household income was categorized as follows: 0.00, 0.01–1.00, 1.01–2.00, 2.01–3.00, and ≥3.01 million yen. The exchange rate for the United States (USA) dollars and Japanese yen used was that of 29 November 2016; one US dollar was the equivalent of 112.31 Japanese yen.

### 2.5. Other Categorization of the Data

Subjects were divided into eight age categories: 0–8, 9–16, 17–29, 30–39, 40–49, 50–59, 60–69, and 70–74 years. Subjects were also categorized based on the number of household members: 1 or 2, 3, and ≥4. Additionally, the data for six residential areas (Chuo, Hanamigawa, Inage, Wakaba, Midori, and Mihama) in Chiba City as of 2 April 2014, were provided.

### 2.6. Statistical Analysis

For categorical variables, chi-square tests and Cochran–Armitage trend tests were performed for univariate analysis. For continuous variables such as age and the number of days for which dental services were provided, means, and standard deviations (SDs) were calculated, and analysis of variance and the general linear model for trend tests were performed.

The association between equivalent household income and access to dental care services was evaluated by using the generalized estimating equation (GEE) model [15]. We defined households as clusters; because utilization for dental services was binary, we set up the distribution to binominal and the link function to logit. The number of days spent on the dental services was count data; thus, we set up the distribution to Poisson and the link function to log. The analysis of the number of days for which dental services were provided was performed for all individuals who used the services in the year. We evaluated the association between income levels with the presence or absence of dental visits and the number of days spent for the services using a model including sex, equivalent household income, interaction between sex and equivalent income, age, number of household members, and residential areas. Since a previous study reported a significant difference for the impact of SES on health outcomes between men and women, the interaction of both sexes was included in the model [16]. The tests for the homogeneity of the slopes between men and women were performed; the null hypothesis tested was that of no difference in linear trend coefficients for the equivalent income category between men and women. Subsequently, in a subgroup analysis, we examined the association between SES and health outcomes in each age group, as the percentage of self-paid medical fees and financial support is age-dependent.

A *p*-value less than 0.05 was considered statistically significant. All statistical analyses were performed using SAS software version 9.4 (SAS Institute, Cary, NC, USA).

### 2.7. Sensitivity Analysis

As the association between the income and access to the dental care could depend on the diseases, we evaluated it using two encoded disease names: dental caries, and gingivitis and periodontal disease. According to the Table of International Classification of Disease for the Use of Social Insurance established by the Ministry of Health, Labour and Welfare in Japan [17], the insurance claims for dental caries were encoded as “1101,” which is equivalent to K02 of International Statistical Classification of Diseases and Related Health Problems 10th revision (ICD-10) [18], and those for gingivitis and periodontal disease were encoded as “1002,” equivalent to K05. The individuals with claims of “1101” were defined as users of insurance for dental caries care and those of “1102” were defined as users of insurance for gingivitis and periodontal disease care. The numbers of days spent on the services of each encoded disease were the total sum of days required for service for the codes of “1101” and “1102”, respectively.

## 3. Results

A total of 216,211 (110,942 (51%) women) beneficiaries of NHI in Chiba City, Japan, were enrolled on the insurance from 1 April 2014 to 31 March 2015. A total of 4,048,547 claims were submitted during the period, 7350 (0.18%) of which were excluded as they contained no individual identification information. Of the remaining 4,041,197 claims, the 497,900 (12.3%) were dental care claims.

Characteristics of beneficiaries and access to dental care services stratified by income levels are shown in Table 1. Approximately 50.3% of the beneficiaries were users of dental care services, and the average number of days spent on dental care services (SD) was 7.7 (7.1).

**Figure 1:**
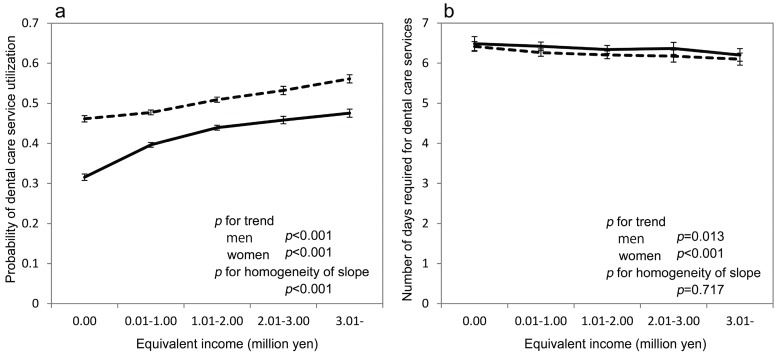
(**a**) Probability of dental care service utilization and (**b**) mean number of days required for dental care services predicted using the generalized estimating equation based on equivalent income category from 1 April 2014 to 31 March 2015. The solid lines represent men and dotted lines represent women. Estimated probability and 95% confidence intervals are shown. The analysis of number of days required for dental care services included 108,689 beneficiaries who received dental care services from 1 April 2014 to 31 March 2015. Sex, equivalent income, age, residential area, number of family members, and the interaction between sex and equivalent income were included in the models. *p*-values for the linear trends for equivalent income categories for each sex were calculated. *p*-values for the test for homogeneity of slope, in which the null hypothesis was that there was no difference of linear trend coefficients for the equivalent income categories between men and women, were also calculated.

Significant positive linear trends existed, indicating that the subjects with lower income were less likely to use dental care services, for both sexes (*p* < 0.001) (Figure 1a). In addition, the gradients were steeper in men than in women (*p* < 0.001). The probability of the utilization (95% confidence interval (CI)) estimated by GEE (generalized estimating equation) in the lowest and highest income groups were 0.316 (0.308–0.324) and 0.476 (0.465–0.486) in men and 0.462 (0.454–0.469) and 0.561 (0.551–0.572) in women, respectively. However, a significant inverse association between the income and number of days required for dental services for both men (*p* = 0.013) and women (*p* < 0.001) (Figure 1b) was observed. The tests for the homogeneity of the slope between men and women were not significant (*p* = 0.717). Mean number of days (95% CI) estimated by GEE for the lowest and highest income groups were 6.49 (6.31–6.66) and 6.20 (6.05–6.36) for men and 6.42 (6.30–6.54) and 6.10 (5.95–6.25) days for women, respectively. Coefficients estimated by GEE for the association of equivalent income with the utilization of dental care services and the number of days for which dental services were required are shown in Table S1.

**Figure 2:**
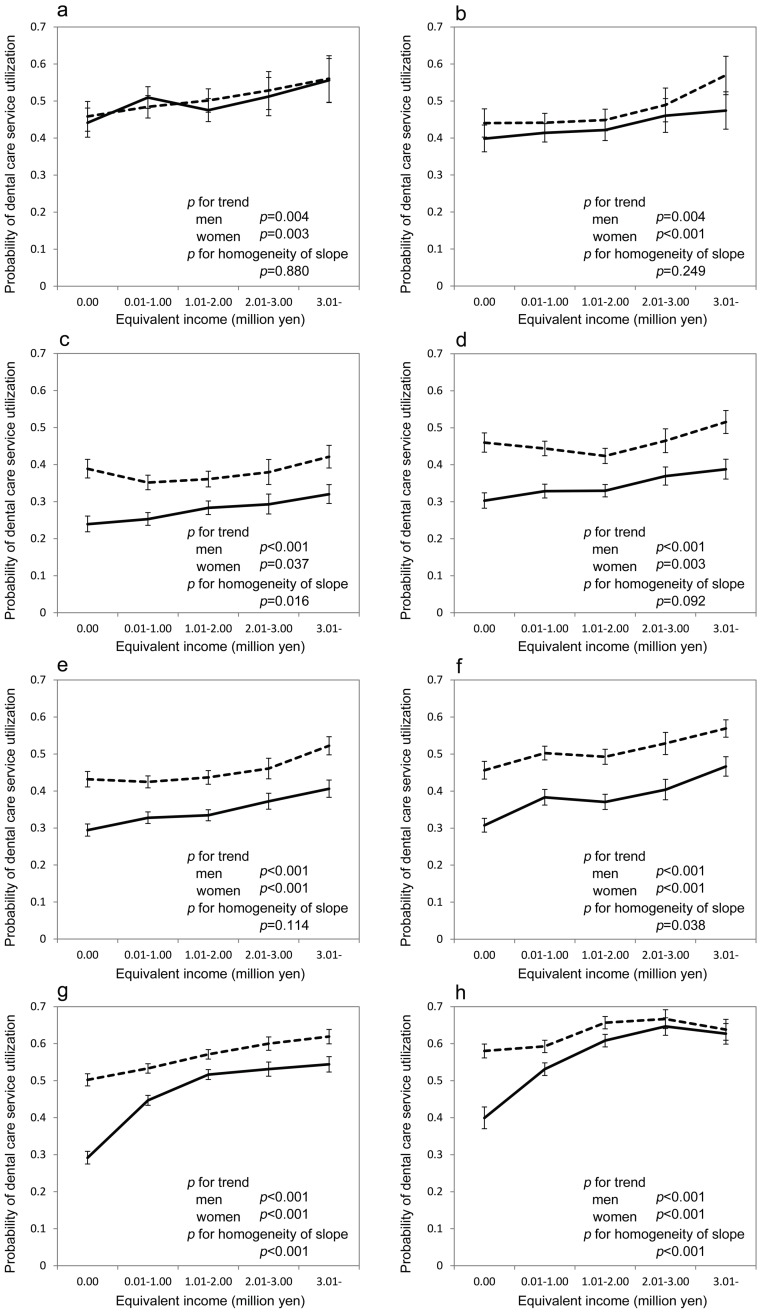
Probability of all dental care utilization predicted using the generalized estimating equation for each age group based on equivalent income category from 1 April 2014 to 31 March 2015. (**a**) 0–8 years (*n* = 7320); (**b**) 9–16 years (*n* = 9075); (**c**) 17–29 years (*n* = 16,893); (**d**) 30–39 years (*n* = 18,907); (**e**) 40–49 years (*n* = 26,434); (**f**) 50–59 years (*n* = 21,285); (**g**) 60–69 years (*n* = 67,344); (**h**) 70–74 years (*n* = 48,953). Solid lines represent men and dotted lines represent women. Estimated probabilities and 95% confidence intervals are shown. Sex, equivalent income, residential area, number of family members, and the interaction between sex and equivalent income were included in the models. *p*-values for the linear trends for equivalent income categories for each sex were calculated. *p*-values for the test of the homogeneity of slope, in which null hypothesis was that there was no difference in the linear trend coefficients for the equivalent income categories between men and women, were also calculated.

The results of subgroup analysis for utilization of dental services by age groups are shown in Figure 2. The significant positive linear associations between income and utilization for dental care services were observed in all age groups for both sexes. Furthermore, the gradient was significantly steeper in men than in women, particularly for the elderly beneficiaries (*p* < 0.001 for both age groups of 60–69 years and 70–74 years as shown in Figure 2g,h). The association between income and number of days required for dental care services in each age group is shown in Figure S1. Significant inverse associations between income and number of days required for dental services for various age groups dependent on sex (e.g., both sexes in Figure S1g) were observed.

The results of the sensitivity analyses are shown in Figures S2 and S3; the associations measured here were similar to those for dental care services as a whole.

## 4. Discussion

Our study verifies the existence of income-related inequalities in access to dental care services among all age groups for both sexes in Japan. Socioeconomic inequality in the access to dental care services has been confirmed in a number of countries [6,7,8,9,10,11,19,20,21], while the influence of country-specific health insurance systems has also been suggested [8,22,23]. Therefore, we think it would be meaningful and interesting to clarify the impact of SES (socioeconomic status) on the access to dental care services in Japan, where a universal insurance system has been established. So far, only one study evaluating the association between income levels and dental care service utilization existed in Japan; it showed that the income-related inequality is present only for preventive dental care in men but not for curative dental care services for both sexes [7]. In contrast, our study design had several advantages such as a large sample size (including a wide range of age groups), no missing data in principle, and data accuracy. In fact, Hirai et al. indicated that a low-income group tended to poorly respond to the mailed self-administrated questionnaire. They showed a significant association between income and mortality in all subjects with governmental income data, but not with the data obtained using questionnaires [24]. Our study used local government administrative data regarding both the subject’s income and their dental care access to avoid any bias due to a lack of responses and uncertain data. We demonstrated a significant association between lower income and decreased access to dental care services in all age groups including children, adolescents, and the elderly. As a result of the universal health insurance system established in 1961 [12], all people living in Japan were covered by the public health insurance during this study and were therefore eligible to have medical care including dental care, at a subsidized price (10–30% of the total cost of treatment). In addition, many Japanese municipalities provided their own medical support for children. The fact that income-related inequality was found to exist regardless of subsidized public health insurance prompted us to think measures other than financial support would be necessary to rectify this inequity. Other factors that could cause the income-related inequality, such as low knowledge of health, deficient health literacy [25], and difficulty managing time for consultations due to busy lifestyles [26], have been described among low-income groups.

A study by Matsuyama et al. showed that the poorest people in Japan used dental prosthesis as frequently as the highest income people in Japan did, although low-income people in general tended not to use the prosthesis [2]. A possible explanation for this puzzling pattern was that the social welfare benefits for the poorest individuals encouraged dental care service utilization. In fact, the people who receive public assistance, called “Seikatsu-hogo”, can receive medical and dental treatment free of charge. As people with public assistance were not included in our study, we could not evaluate the effect of public assistance on access to dental care services. Although free dental care has been reported as a possible effective measure to eliminate inequity in access to dental care services [2], providing an aggressive and free national medical policy is not realistic in modern Japanese society, given the country’s current financial status.

Our study also revealed that the association between income and dental care utilization was more evident in men compared to women, in accordance with previous reports [7]. One possible explanation could be that men are more susceptible to low income than women. In fact, Sakurai et al. claimed that lower household income was associated with a higher risk for psychological distress in men but not in women [16]. Furthermore, a study based in the United States showed that women had a greater interest in health and sought health care services more frequently than men [27]. The greater interest in health and literacy observed among women might alleviate the impact of low income in access to the services. Additionally, our subgroup analysis indicated a greater impact of income on the utilization in men compared to women, especially in elderly groups. In Japan, consistent lower attention to oral health has been reported among working men than working women; men might be too busy to allocate time to their oral health [28]. Indeed, our results showed that men are less likely to use dental care services compared to women throughout the working age. After retirement, however, men might visit dentists at earlier stages of dental diseases. Even so, this behavioral change might not occur in poor men, whose social situation likely stays the same regardless of age. As a result, the gap in utilization between men and women in elderly populations diminished in the higher income groups, but not in lower income groups.

The current study showed a significant association between a greater number of days spent on dental care services and lower income level. A previous study suggested that lower income patients tend to have minimum and insufficient dental care, while higher income groups have exhaustive dental care [29]. The identified opposite association in this study, though modest, could indicate that more severe oral conditions existed among lower income groups at the time of the dental visit in Japan.

We conducted a sensitivity analysis to examine the association between income level and the access to curative dental care for dental caries, and gingivitis and periodontal disease, independently. We found that the associations between income levels and access were similar to that of the provision of all dental care services as a whole. This suggests that the impact of income on the utilization and number of days required for dental care services did not depend on the type of dental care provided.

Our study has several limitations. Generalizability of our results would be limited as our target subjects were the beneficiaries of the NHI only, and we did not include those with public assistance or with other public insurances. In other words, employees and their family members were not included as our subjects. Based on the reports in Chiba City, the percentages of NHI beneficiaries to the total population in 2013 were 18.3% for individuals aged 0–39 years, 20.4% aged 40–54 years, 37.0% aged 55–64 years, and 77.9% aged 65–74 years. Thus, this study has low generalizability especially in the working age group. Furthermore, subjects were only included if they were residents of Chiba City, a satellite, urban city of Tokyo, where relatively abundant medical institutions and dental clinics exist. The associations measured thus could be different in rural areas.

## 5. Conclusions

We revealed the existence of income-related inequality in access to dental care services in all age groups for both sexes in Japan using data from individual’s incomes declared for taxation and insurance claims. The universal health insurance system in Japan and the medical support provided for infants and children in Chiba City could not eliminate this inequality.

## Figures and Tables

**Table 1:** Subjects’ characteristics and description of access to dental care services from 1 April 2014 to 31 March 2015.

Variables	Equivalent Income (Million Yen) ^3^							
	All	0.00	0.01–1.00	1.01–2.00	2.01–3.00	≥3.01	*p*-Value	*p*-Value for Trend
Number of subjects	216,211	37,918	70,575	63,303	23,162	21,253		
Men (%) ^1^	105,269 (48.7)	16,674 (44.0)	33,083 (46.9)	32,292 (51.0)	12,553 (54.2)	10,667 (50.2)	<0.001	<0.001
Age ^2^	53.0 (19.8)	51.4 (19.3)	54.6 (20.0)	54.1 (19.9)	51.3 (19.6)	49.6 (18.7)	<0.001	<0.001
**Number of Family Members ^1^**								
1 or 2 (%)	154,222 (71.3)	32,494 (85.7)	49,698 (70.4)	43,133 (68.1)	152,04 (65.6)	13,693 (64.4)	<0.001	
3 (%)	35,299 (16.3)	3260 (8.6)	12,547 (17.8)	11,120 (17.6)	4472 (19.3)	3900 (18.4)		
4 and more	26,690 (12.3)	2164 (5.7)	8330 (11.8)	9050 (14.3)	3486 (15.1)	3660 (17.2)		
**Residual Area ^1^**								
Chuo (%)	43,349 (20.1)	8691 (22.9)	13,577 (19.2)	11,883 (18.8)	4702 (20.3)	4496 (21.2)	<0.001	
Hanamigawa (%)	40,345 (18.7)	6950 (18.3)	13,449 (19.1)	11,801 (18.6)	4160 (18.0)	3985 (18.8)		
Inage (%)	34,699 (16.1)	5854 (15.4)	11,087 (15.7)	10,396 (16.4)	3697 (16.0)	3665 (17.2)		
Wakaba (%)	40,584 (18.8)	7669 (20.2)	13,026 (18.5)	11,835 (18.7)	4449 (19.2)	3605 (17.0)		
Midori (%)	25,645 (11.9)	3857 (10.2)	8497 (12.0)	7805 (12.3)	2852 (12.3)	2634 (12.4)		
Mihama (%)	31,589 (14.6)	4897 (12.9)	10,939 (15.5)	9583 (15.1)	3302.(14.3)	2868 (13.5)		
Users of dental care services ^1^	108,689 (50.3)	16,471 (43.4)	35,304 (50.0)	33,418 (52.8)	12,173 (52.6)	11,323 (53.3)	<0.001	<0.001
The number of days for dental care services ^2^	7.7 (7.1)	7.8 (7.2)	7.8 (7.2)	7.7 (7.0)	7.5 (7.0)	7.3 (6.8)	<0.001	<0.001

^1^ Number of beneficiaries (percent). ^2^ Mean (standard deviation). ^3^ As of 29 November 2016, 1 US dollar was equivalent to 112.31 Japanese yen.

## Supplementary Materials

The following are available online at www.mdpi.com/1660-4601/14/5/524/s1. Figure S1: Number of days for all dental care services predicted using the generalized estimating equation for each age group based on equivalent income category from 1 April 2014 to 31 March 2015. (a) 0–8 years (*n* = 3671); (b) 9–16 years (*n* = 3970); (c) 17–29 years (*n* = 5355); (d) 30–39 years (*n* = 7212); (e) 40–49 years (*n* = 10,107); (f) 50–59 years (*n* = 9340); (g) 60–69 years (*n* = 37,616); (h) 70–74 years (*n* = 31,418). Solid lines represent men and dotted lines represent women. The estimated number of days in which dental services were required and the 95% confidence intervals are shown. The analysis included 108,689 beneficiaries who received dental care services from 1 April 2014 to 31 March 2015. Sex, equivalent income, residential area, number of family members, and the interaction between sex and equivalent income were included in the models. *p*-values for the linear trends for equivalent income categories for each sex were calculated. *p*-values for the test for the homogeneity of the slope, in which null hypothesis was that there was no difference in linear trend coefficients for the equivalent income category between men and women, were also calculated. Figure S2: Probability of utilization and the mean number of days for treatment of dental caries predicted using the generalized estimating equation. Solid lines represent men and dotted lines represent women. The estimated probability and 95% confidence intervals are shown. The analysis of the number of days required for the treatment of dental caries included 58,827 persons who received treatment of dental caries from 1 April 2014 to 31 March 2015. Sex, equivalent income, age, residential area, number of family members, and the interaction between sex and equivalent income were included in the models. *p*-values for the linear trend for the equivalent income categories in each sex were calculated, and *p*-values for the tests of homogeneity of the slope, for which the null hypothesis was that there was no difference in the linear trend coefficients for equivalent income category between men and women, were also calculated. Figure S3: Probability of utilization and mean number of days for treatment of gingivitis and periodontal disease predicted using the generalized estimating equation. Solid lines represent men and dotted lines represent women. The estimated probability and 95% confidence intervals are shown. The analysis of the number of days required for the treatment of dental caries included 52,783 persons who received treatment of gingivitis and periodontal disease from 1 April 2014 to 31 March 2015. Sex, equivalent income, age, residential area, number of family members, and the interaction between sex and equivalent income were included in the models. *p*-values for the linear trend for the equivalent income category in each sex were calculated, and *p*-values for the test of homogeneity of the slope, in which the null hypothesis was that there was no difference in the linear trend coefficients for equivalent income category between men and women, were also calculated. Table S1: Coefficients estimated using the generalized estimating equation for the association of equivalent income with dental care utilization and number of days spent for the services.

## References

[1] Haugejorden O., Klock K.S., Astrom A.N., Skaret E., Trovik T.A. (2008). Socio-economic inequality in the self-reported number of natural teeth among Norwegian adults—An analytical study. Community Dent. Oral Epidemiol..

[2] Matsuyama Y., Aida J., Takeuchi K., Tsakos G., Watt R.G., Kondo K., Osaka K. (2014). Inequalities of dental prosthesis use under universal healthcare insurance. Community Dent. Oral Epidemiol..

[3] Schwendicke F., Dorfer C.E., Schlattmann P., Page L.F., Thomson W.M., Paris S. (2015). Socioeconomic inequality and caries: A systematic review and meta-analysis. J. Dent. Res..

[4] Pattussi M.P., Peres K.G., Boing A.F., Peres M.A., da Costa J.S.D. (2010). Self-rated oral health and associated factors in Brazilian elders. Community Dent. Oral Epidemiol..

[5] Wamala S., Merlo J., Bostrom G. (2006). Inequity in access to dental care services explains current socioeconomic disparities in oral health: The Swedish National surveys of Public Health 2004–2005. J. Epidemiol. Community Health.

[6] Grytten J., Holst D., Skau I. (2012). Demand for and utilization of dental services according to household income in the adult population in Norway. Community Dent. Oral Epidemiol..

[7] Murakami K., Aida J., Ohkubo T., Hashimoto H. (2014). Income-related inequalities in preventive and curative dental care use among working-age Japanese adults in urban areas: A cross-sectional study. BMC Oral Health.

[8] Listl S. (2011). Income-related inequalities in dental service utilization by Europeans aged 50+. J. Dent. Res..

[9] Hjern A., Grindefjord M., Sundberg H., Rosen M. (2001). Social inequality in oral health and use of dental care in Sweden. Community Dent. Oral Epidemiol..

[10] Larson K., Halfon N. (2010). Family income gradients in the health and health care access of US children. Matern. Child. Health J..

[11] Tchicaya A., Lorentz N. (2014). Socioeconomic inequalities in the non-use of dental care in Europe. Int. J. Equity Health.

[12] Ikegami N., Yoo B.K., Hashimoto H., Matsumoto M., Ogata H., Babazono A., Watanabe R., Shibuya K., Yang B.M., Reich M.R. (2011). Japanese universal health coverage: Evolution, achievements, and challenges. Lancet.

[13] Fujita M., Sato Y., Nagashima K., Takahashi S., Hata A. (2016). Income related inequality of health care access in Japan: A retrospective cohort study. PloS ONE.

[14] Organisation for Economic Co-Operation and Development Terms of Reference OECD Project on the Distribution of Household Incomes 2014/15 Collection. http://www.oecd.org/statistics/data-collection/Income%20distribution_guidelines.pdf.

[15] Hanley J.A., Negassa A., deB Edwardes M.D., Forrester J.E. (2003). Statistical analysis of correlated data using generalized estimating equations: An orientation. Am. J. Epidemiol..

[16] Sakurai K., Kawakami N., Yamaoka K., Ishikawa H., Hashimoto H. (2010). The impact of subjective and objective social status on psychological distress among men and women in Japan. Soc. Sci. Med..

[17] Ministry of Health, Labour and Welfare Table of International Classification of Diseases for the Use of Social Insurance. http://www.mhlw.go.jp/bunya/iryouhoken/database/zenpan/shobyo_bunrui.html.

[18] World Health Organization International Statistical Classiﬁcation of Diseases and Related Health Problems, 10th Revision-WHO Version for 2016. http://apps.who.int/classifications/icd10/browse/2016/en.

[19] Vikum E., Krokstad S., Holst D., Westin S. (2012). Socioeconomic inequalities in dental services utilisation in a Norwegian county: The third Nord-Trondelag Health Survey. Scand. J. Public Health.

[20] Somkotra T., Detsomboonrat P. (2009). Is there equity in oral healthcare utilization: Experience after achieving Universal Coverage. Community Dent. Oral Epidemiol..

[21] Raittio E., Kiiskinen U., Helminen S., Aromaa A., Suominen A.L. (2015). Income-related inequality and inequity in the use of dental services in Finland after a major subsidization reform. Community Dent. Oral Epidemiol..

[22] Palència L., Espelt A., Cornejo-Ovalle M., Borrell C. (2014). Socioeconomic inequalities in the use of dental care services in Europe: What is the role of public coverage?. Community Dent. Oral Epidemiol..

[23] Elstad J.I. (2017). Dental care coverage and income-related inequalities in foregone dental care in Europe during the great recession. Community Dent. Oral Epidemiol..

[24] Hirai H., Kondo K., Kawachi I. (2012). Social Determinants of Active Aging: Differences in Mortality and the Loss of Healthy Life between Different Income Levels among Older Japanese in the AGES Cohort Study. Curr. Gerontol. Geriatr. Res..

[25] Macek M.D., Haynes D., Wells W., Bauer-Leffler S., Cotton P.A., Parker R.M. (2010). Measuring conceptual Health knowledge in the context of oral health literacy: Preliminary results. J. Public Health Dent..

[26] Kullgren J.T., McLaughlin C.G., Mitra N., Armstrong K. (2012). Nonfinancial barriers and access to care for U.S. adults. Health Serv. Res..

[27] Green C.A., Pope C.R. (1999). Gender, psychosocial factors and the use of medical services: A longitudinal analysis. Soc. Sci. Med..

[28] Kawamura M., Allan F., Wright C., Sasahara H., Yamasaki Y., Suh S., Iwamoto Y. (1999). An analytical study on gender differences in self-reported oral health care and problems of Japanese employees. J. Occup. Health.

[29] Widström E., Seppälä T. (2012). Willingness and ability to pay for unexpected dental expenses by Finnish adults. BMC Oral Health.

