# Supplementary material for: Income-Related Inequalities in Access to Dental Care Services in Japan
Source: Int J Environ Res Public Health. 2017 May 12;14(5):524. doi: 10.3390/ijerph14050524 (PMC5451975; doi:10.3390/ijerph14050524)
Supplement: Supplementary file 1 [file ijerph-14-00524-s001.pdf]

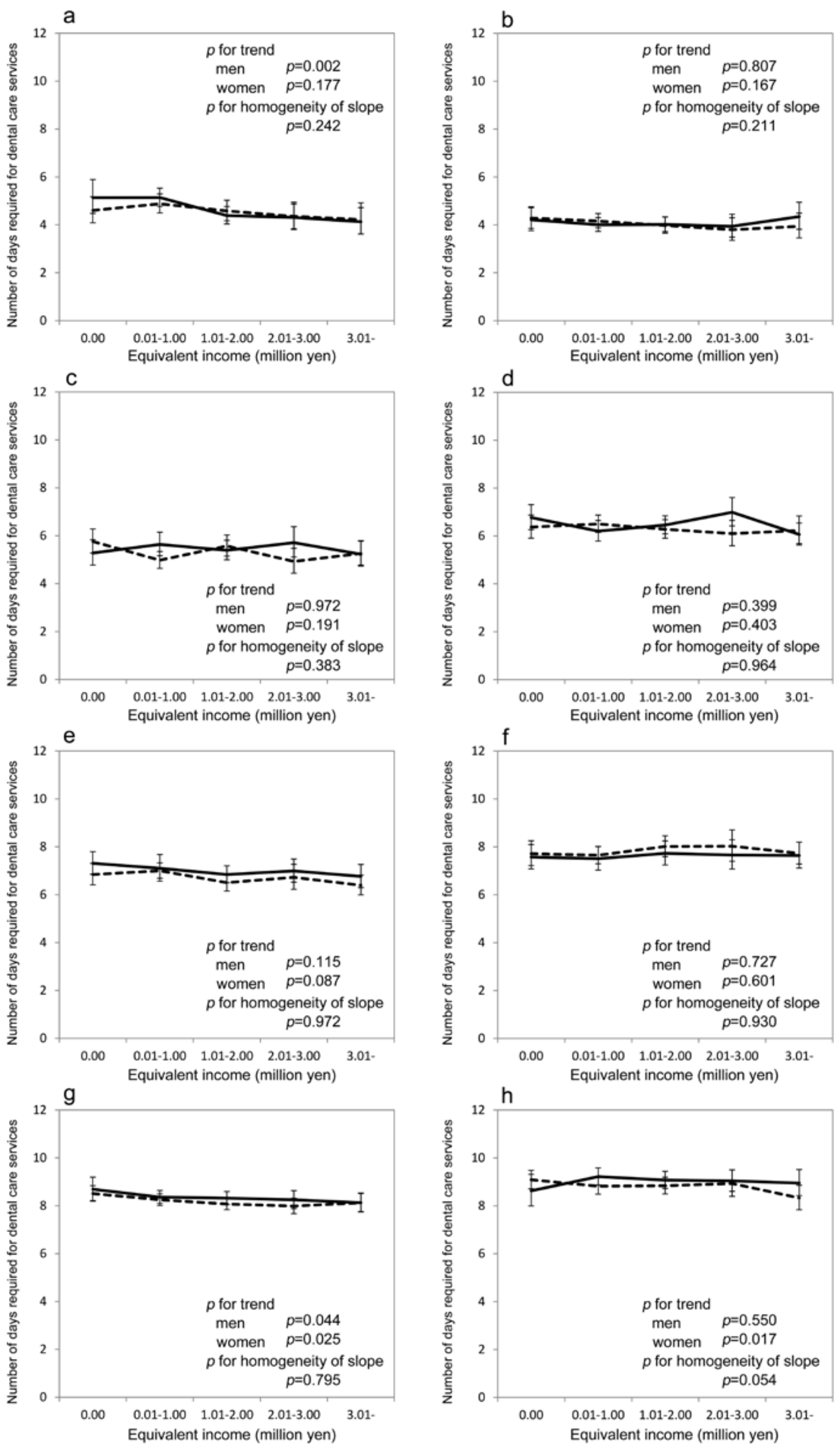

Figure S1. Number of days for all dental care services predicted using the generalized estimating equation for each age group based on equivalent income category from 1 April 2014 to 31 March 2015. **a**: 0–8 years (*n* = 3671); **b**: 9–16 years (*n* = 3970); **c**: 17–29 years (*n* = 5355); **d**: 30–39 years (*n* = 7212); **e**: 40–49 years (*n* = 10,107); **f**: 50–59 years (*n* = 9340); **g**: 60–69 years (*n* = 37,616); **h**: 70–74 years (*n* = 31,418). Solid lines represent men and dotted lines represent women. The estimated number of days in which dental services were required and the 95% confidence intervals are shown. The analysis included 108,689 beneficiaries who received dental care services from 1 April 2014 to 31 March 2015. Sex, equivalent income, residential area, number of family members, and the interaction between sex and

equivalent income were included in the models. p-values for the linear trends for equivalent income categories for each sex were calculated. p-values for the test for the homogeneity of the slope, in which null hypothesis was that there was no difference in linear trend coefficients for the equivalent income category between men and women, were also calculated.

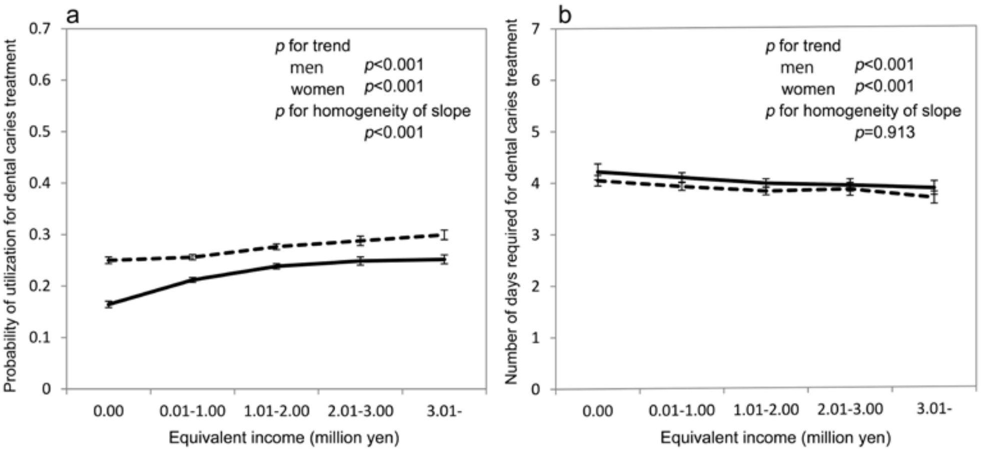

Figure S2. Probability of utilization and the mean number of days for treatment of dental caries predicted using the generalized estimating equation. Solid lines represent men and dotted lines represent women. The estimated probability and 95% confidence intervals are shown. The analysis of the number of days required for the treatment of dental caries included 58,827 persons who received treatment of dental caries from 1 April 2014 to 31 March 2015. Sex, equivalent income, age, residential area, number of family members, and the interaction between sex and equivalent income were included in the models. p-values for the linear trend for the equivalent income categories in each sex were calculated, and p-values for the tests of homogeneity of the slope, for which the null hypothesis was that there was no difference in the linear trend coefficients for equivalent income category between men and women, were also calculated.

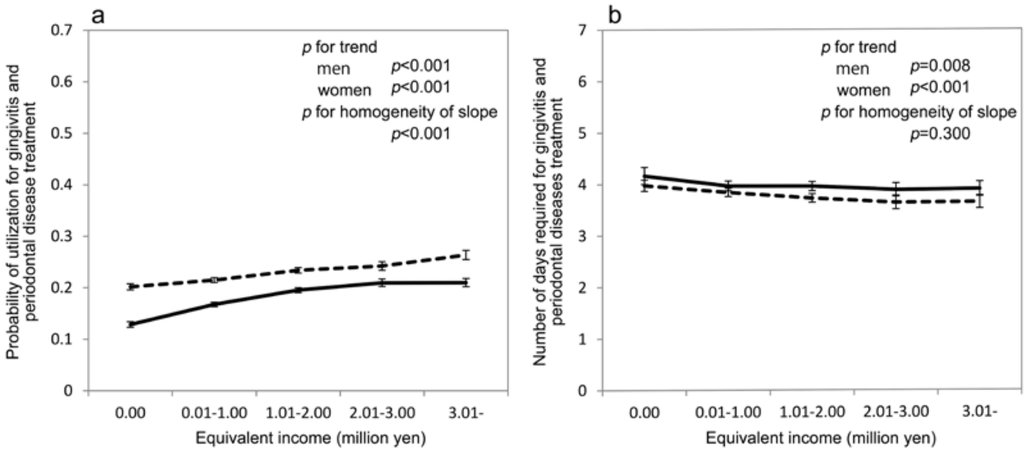

Figure S3. Probability of utilization and mean number of days for treatment of gingivitis and periodontal disease predicted using the generalized estimating equation. Solid lines represent men and dotted lines represent women. The estimated probability and 95% confidence intervals are shown. The analysis of the number of days required for the treatment of dental caries included 52,783 persons who received treatment of gingivitis and periodontal disease from 1 April 2014 to 31 March 2015. Sex, equivalent income, age, residential area, number of family members, and the interaction between sex and equivalent income were included in the models. p-values for the linear trend for the equivalent income category in each sex were calculated, and p-values for the test of homogeneity of the slope, in which the null hypothesis was that there was no difference in the linear trend coefficients for equivalent income category between men and women, were also calculated.

Table S1. Coefficients estimated using the generalized estimating equation for the association of equivalent income with dental care utilization and number of days spent for the services.

Variables	Dental Care Utilization [1]			Number of Days Spent for Dental Care Services [2]		
	Coefficients	95% Confidence Interval	*p*-Value	Coefficients	95% Confidence Interval	*p*-Value
**Age (Year)**						
0–8	−0.56	−0.61,−0.50	<0.001	−0.61	−0.65,−0.57	<0.001
9–16	−0.78	−0.83,−0.73	<0.001	−0.76	−0.79,−0.72	<0.001
17–29	−1.28	−1.32,−1.24	<0.001	−0.48	−0.51,−0.44	<0.001
30–39	−1.01	−1.05,−0.97	<0.001	−0.30	−0.32,−0.27	<0.001
40–49	−1.00	−1.03,−0.96	<0.001	−0.23	−0.25,−0.20	<0.001
50–59	−0.79	−0.83,−0.76	<0.001	−0.11	−0.13,−0.09	<0.001
60–69	−0.37	−0.39,−0.34	<0.001	−0.07	−0.09,−0.06	<0.001
70–74	0.00	reference		0.00	reference	
**Number of Family Members**						
1 or 2	0.05	0.01,0.08	0.013	0.03	0.01,0.06	0.006
3	−0.03	−0.07,0.01	0.150	0.03	0.01,0.06	0.014
4 and more	0.00	reference		0.00	reference	
**Residual Area**						
Chuo	−0.07	−0.10,−0.04	<0.001	0.00	−0.02,0.02	0.751
Hanamigawa	−0.06	−0.09,−0.03	<0.001	0.02	0.00,0.04	0.019
Inage	0.01	−0.02,0.05	0.487	-0.02	−0.04,0.00	0.047
Wakaba	−0.14	−0.17,−0.10	<0.001	-0.03	−0.05,−0.01	0.004
Midori	−0.02	−0.06,0.01	0.243	-0.04	−0.06,−0.02	<0.001
Mihama	0.00	reference		0.00	reference	
**Sex**						
Men	−0.34	−0.40,−0.29	<0.001	0.02	−0.02,0.05	0.317
Women	0.00	reference		0.00	reference	
**Equivalent Income (Million Yen) [3]**						
0.00	−0.40	−0.45,−0.35	<0.001	0.05	0.02,0.08	<0.001
0.01–1.00	−0.34	−0.38,−0.29	<0.001	0.03	0.00,0.05	0.055
1.01–2.00	−0.21	−0.26,−0.16	<0.001	0.02	−0.01,0.05	0.214
2.01–3.00	−0.12	−0.17,−0.06	<0.001	0.01	−0.02,0.05	0.469
3.01–	0.00	reference		0.00	reference	
**Sex * Equivalent Income (Million Yen)**						
Men * 0.00	−0.27	−0.34,−0.21	<0.001	−0.01	−0.05,0.04	0.791
Men * 0.01–1.00	0.01	−0.05,0.08	0.632	0.01	−0.03,0.05	0.687
Men * 1.01–2.00	0.06	0.00,0.13	0.042	0.00	−0.03,0.04	0.816
Men * 2.01–3.00	0.05	−0.03,0.12	0.199	0.01	−0.03,0.06	0.562

[1] The number of subjects was 216,211. [2] The analysis included 108,689 beneficiaries who received dental care services from April 1, 2014, through March 31, 2015. [3] As of November 29, 2016, 1 US dollar was equivalent to 112.31 Japanese yen.
